# Ca^2+^ imbalance caused by ERdj5 deletion affects mitochondrial fragmentation

**DOI:** 10.1038/s41598-021-99980-9

**Published:** 2021-11-02

**Authors:** Riyuji Yamashita, Shohei Fujii, Ryo Ushioda, Kazuhiro Nagata

**Affiliations:** 1 Laboratory of Molecular and Cellular Biology, Faculty of Life Sciences, Kyoto Sangyo University, Kyoto, 603-8555 Japan; 2 Institute for Protein Dynamics, Kyoto Sangyo University, Kyoto, 605-8555 Japan; 3 JT Biohistory Research Hall, Murasaki Town 1-1, Takatsuki City, Osaka 569-1125 Japan

**Keywords:** Endoplasmic reticulum, Endoplasmic reticulum

## Abstract

The endoplasmic reticulum (ER) is the organelle responsible for the folding of secretory/membrane proteins and acts as a dynamic calcium ion (Ca^2+^) store involved in various cellular signalling pathways. Previously, we reported that the ER-resident disulfide reductase ERdj5 is involved in the ER-associated degradation (ERAD) of misfolded proteins in the ER and the activation of SERCA2b, a Ca^2+^ pump on the ER membrane. These results highlighted the importance of the regulation of redox activity in both Ca^2+^ and protein homeostasis in the ER. Here, we show that the deletion of ERdj5 causes an imbalance in intracellular Ca^2+^ homeostasis, the activation of Drp1, a cytosolic GTPase involved in mitochondrial fission, and finally the aberrant fragmentation of mitochondria, which affects cell viability as well as phenotype with features of cellular senescence. Thus, ERdj5-mediated regulation of intracellular Ca^2+^ is essential for the maintenance of mitochondrial homeostasis involved in cellular senescence.

## Introduction

The endoplasmic reticulum (ER) serves as the site of folding for newly synthesized secretory and membrane proteins^1,2^, and various molecular chaperones and oxidoreductases function in the folding of nascent polypeptides entering the ER. Since approximately one-third of the total cellular proteins are produced in the ER^3^, protein quality control is essential for normal cellular functions as well as the maintenance of ER homeostasis. Previously, we found a novel disulfide reductase residing in the ER, ERdj5, which contains a J domain at its N-terminus and four thioredoxin-like domains harbouring redox-active Cys-X-X-Cys (CXXC) motifs^4^. ERdj5 reduces disulfide bonds within aberrantly folded proteins, and such substrates are transferred from ERdj5 to Binding Ig Protein (BiP), a member of the HSP70 family of molecular chaperones in the ER, which binds ERdj5 through the His-Pro-Asp (HPD) motif in the J domain. BiP recruits substrates to the retrotranslocation channel for ubiquitin–proteasome-dependent degradation in the cytosol, called ER-associated degradation (ERAD)^5,6^. Thus, ERdj5 is involved in protein quality control via ERAD.

Moreover, the ER plays a role as a reservoir for intracellular Ca^2+^, and the Ca^2+^ concentration in the ER ([Ca^2+^]_ER_) is approximately 10,000 times higher than that in the cytosol^7–9^. A high [Ca^2+^]_ER_ is maintained by sarco/endoplasmic reticulum Ca^2+^-ATPase (SERCA), a calcium pump on the ER membrane that takes up Ca^2+^ from the cytosol into the ER. Thapsigargin (Tg), an inhibitor of SERCA, induces ER stress by decreasing [Ca^2+^]_ER_ due to the inhibition of Ca^2+^ uptake. On the other hand, Ca^2+^ can be released from the ER via inositol 1,4,5-trisphosphate receptors (IP3R)^10^ and ryanodine receptor (RyR)^11,12^, both of which are Ca^2+^ channels on the ER membrane. Ca^2+^ released from the ER acts as a major second messenger within cells and regulates numerous cellular functions, including muscle contraction, cellular motility, vesicular transport, and apoptosis mediated by calcium-binding proteins, including calmodulin and calcineurin, in the cytosol^13,14^.

Redox proteins in the ER are also involved in regulating the activity of calcium pumps and channels localized in the ER membrane, which are important for calcium crosstalk between the ER and cytosol or between the ER and mitochondria. NADPH oxidase 4 (Nox4), a redox protein in the ER, not only inhibits the activity of SERCA by oxidation^15^ but also inactivates IP3R during stress through the production of H_2_O_2_, preventing the influx of calcium from the endoplasmic reticulum into the mitochondria^16^. Glutathione Peroxidase 8 (Gpx8) localizes to the MAM (mitochondria-associated membranes) and inhibits SERCA activity by forming a complex with SERCA^17^, but it is not clear whether this is redox activity dependent or not. Moreover, the Ca^2+^ pump activity of SERCA is regulated by the oxidation and reduction of cysteine on the cytoplasmic and ER luminal sides. There are several cysteine residues on the cytosolic side of SERCA, among which the 649th cysteine (SERCA2) undergoes reversible S-glutathiolation by NO and activates SERCA. On the other hand, in pathological models of atherosclerosis, Cys649 is irreversibly oxidized, which prevents it from undergoing NO-induced S-glutathiolation and inactivates SERCA, which is involved in the progression of atherosclerosis ^18,19^. Regarding redox-mediated regulation of cysteines on the luminal side of the ER, it was previously reported that the activity of SERCA2b, a ubiquitous isoform of SERCA, is negatively regulated by disulfide bond formation between two luminal cysteines of SERCA2b ^20^. Thioredoxin Related Transmembrane Protein 1 (TMX1) is required for the formation of MAM and negatively regulates the Ca^2+^ pumping activity of SERCA2b by forming disulfide bonds at SERCA2b luminal cysteines^21^. Moreover, we reported that ERdj5 activates the pump function of SERCA2b by cleaving its luminal disulfide bridge and that ERdj5 knockout in mouse embryonic fibroblasts (MEFs) caused a decrease in [Ca^2+^]_ER_ due to a reduction in SERCA2b activity^20^. These data suggested that the reductase activity of ERdj5 is involved in Ca^2+^ homeostasis in the ER.

These findings highlighted the importance of redox regulation in the ER by the function of ERdj5 in both protein and Ca^2+^ homeostasis. In ERdj5-deficient mice, sensitivity to ER stress was reported to be dramatically increased in the salivary gland^22^. Recently, another group characterized the phenotypic traits of a Sjögren’s syndrome animal model produced by the knockout of ERdj5^23^. Female mice with Sjögren’s syndrome-like disorder showed inflammatory infiltrates in the salivary gland, serum autoantibodies, reduced saliva secretion, excessive cell death, and deregulated cytokine levels. On the other hand, the downregulation of ERdj5 in cancer cells increased cell death in response to treatment with fenretinide, a cancer chemopreventive and antiproliferative drug^24^.

A putative orthologue of mammalian ERdj5 was reported in *C. elegans* and named DNJ-27. Downregulation of DNJ-27 caused severe paralysis and abnormal mobility in worms that were phenotypically similar to human neurodegenerative diseases. Interestingly, the deletion of DNJ-27 caused aberrant mitochondrial fragmentation in the body wall muscle cells of *C. elegans*^25^, but the detailed mechanisms have never been revealed. In this study, we report that mitochondrial fragmentation was also induced by ERdj5 knockout in mammalian cells. In this process, we succeeded in showing the novel mechanism for the maintenance of transorganellar homeostasis mediated by Ca^2+^ signalling originating from ERdj5.

## Results

### ERdj5 deficiency causes mitochondrial fragmentation in mammalian cells

**Figure 1:**
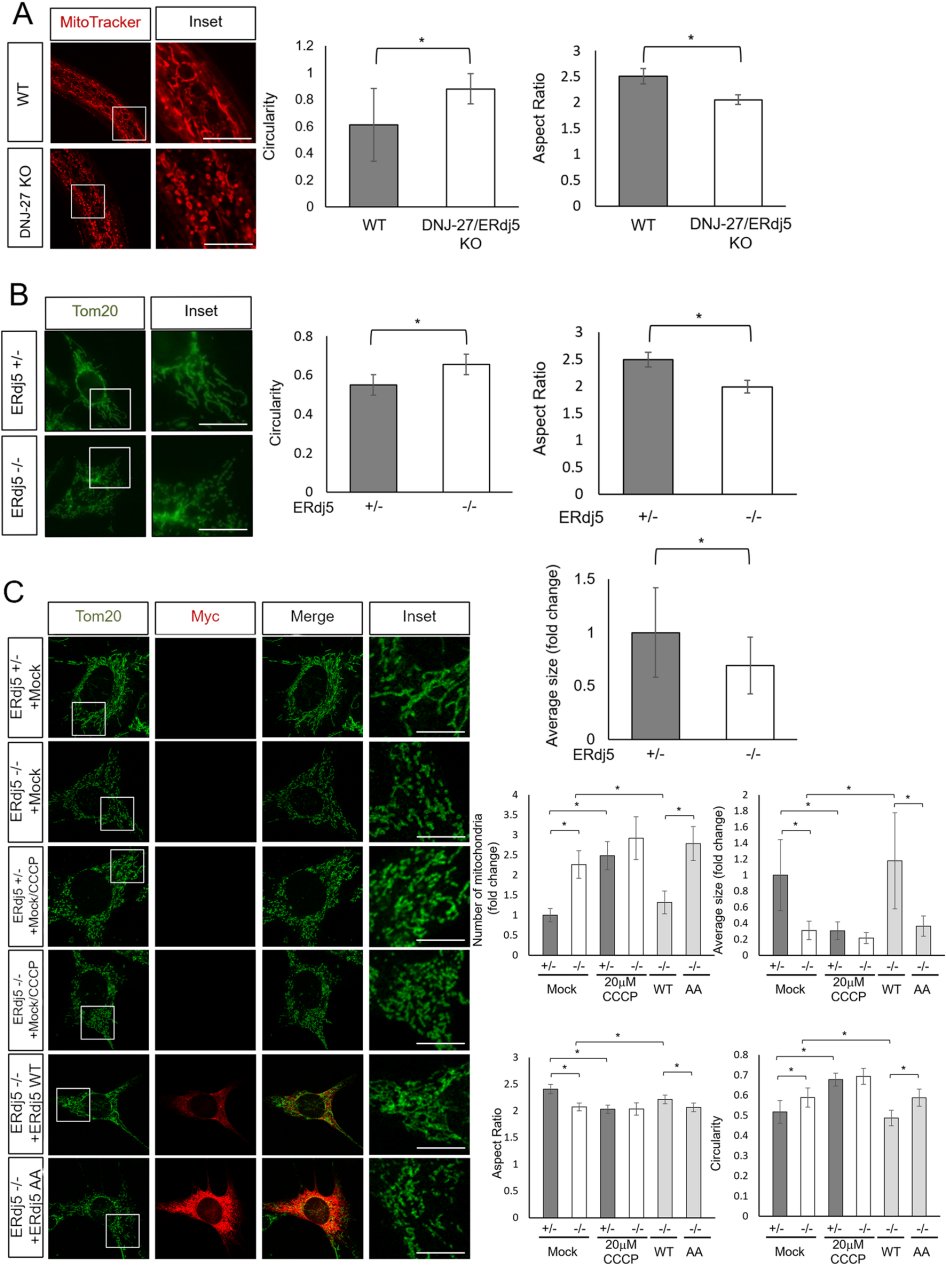
The deletion of ERdj5 caused mitochondrial fragmentation. (**A**) Mitochondrial morphology in WT and DNJ-27/ERdj5-deficient worms. Confocal images of the worms stained with MitoTracker Red CMXRos (red). The right graph shows the circularity and aspect ratio of the mitochondria from MitoTracker Red CMXRos, as analysed by ImageJ. Scale bars = 10 µm. (**B**) Mitochondrial morphology in ERdj5 ± and ERdj5 −/− MEFs. The cells were immunostained with anti-Tom20 antibody (green). The graph shows the circularity, aspect ratio and relative extent of the mitochondria from Tom20, as analysed by ImageJ. Scale bars = 10 µm (**C**) Mitochondrial morphology after the rescue of ERdj5 in ERdj5 −/− cells. Twenty-four hours after transfection of the indicated constructs into MEFs, the cells were incubated in the presence or absence of 20 μM CCCP for 1 h. Then, the cells were immunostained with anti-Tom20 (green) and anti-Myc (red) antibodies. The graphs show the relative extent, number of mitochondria, aspect ratio, and circularity of the mitochondria from Tom20, as analysed by ImageJ. Scale bars = 10 µm (**A-C**) Insets show high-magnification views of the boxed areas. *P < 0.05 by *t* test. The results are reported as the means of 30 worms ± SDs (**A**), 50 cells ± SDs (**B**) or the means of 25 cells ± SDs (**C**).

A previous study demonstrated the protective role of DNJ-27/ERdj5 against the toxicity associated with the expression of human amyloid-β, α-synuclein and polyQ proteins in *C. elegans*^25^. At the same time, the knockdown of DNJ-27/ERdj5 caused mitochondrial fragmentation in body wall muscle cells in *C. elegans*. To confirm the mitochondrial morphology regulated by DNJ-27/ERdj5 in the intestinal cells of *C. elegans*, we obtained DNJ-27/ERdj5-deficient worms (DNJ-27/ERdj5 KO) and observed the mitochondrial structure in WT and DNJ-27/ERdj5 KO worms by MitoTracker Red staining (Fig. 1A). Mitochondrial fragmentation caused by the deletion of DNJ-27/ERdj5 in intestinal cells was observed, suggesting that this mitochondrial fragmentation is not specific to body wall muscle cells. To verify the fragmentation in mammalian cells, we observed mitochondrial morphology in ERdj5-deficient MEFs (Fig. 1B) and HeLa cells in which ERdj5 had been knocked down by small interfering RNA (siRNA) (Fig. S1A). The data showed that the downregulation of ERdj5 caused mitochondrial fragmentation in mammalian cells. Next, we overexpressed ERdj5 in ERdj5-deficient MEFs or HeLa cells in which ERdj5 was knocked down. ERdj5 ± and -/- cells were treated with Carbonylcyanide m-chlorophenylhydrazone (CCCP). CCCP treatment causes mitochondrial fragmentation through the mitochondria membrane depolarization. As a result of CCCP treatment, mitochondrial fragmentation was observed in ERdj5 ± and −/− cells (Fig. 1C). Overexpression of ERdj5/WT-Myc and FLAG completely rescued the changes in mitochondrial structure in ERdj5-deficient MEFs (Fig. 1C ) and knockdown HeLa cells (Fig. S1B and C), whereas overexpression of an ERdj5/AA-Myc mutant in which all CXXC motifs had been converted to Ala-X-X-Ala (AA), generating a reductase activity-null mutant, did not rescue mitochondrial fragmentation (Fig. 1C). These results suggest that ERdj5 is involved in the maintenance of mitochondrial morphology depending on its reductase activity through the CXXC motifs.

### Maintenance of cytosolic calcium homeostasis through ERdj5

**Figure 2:**
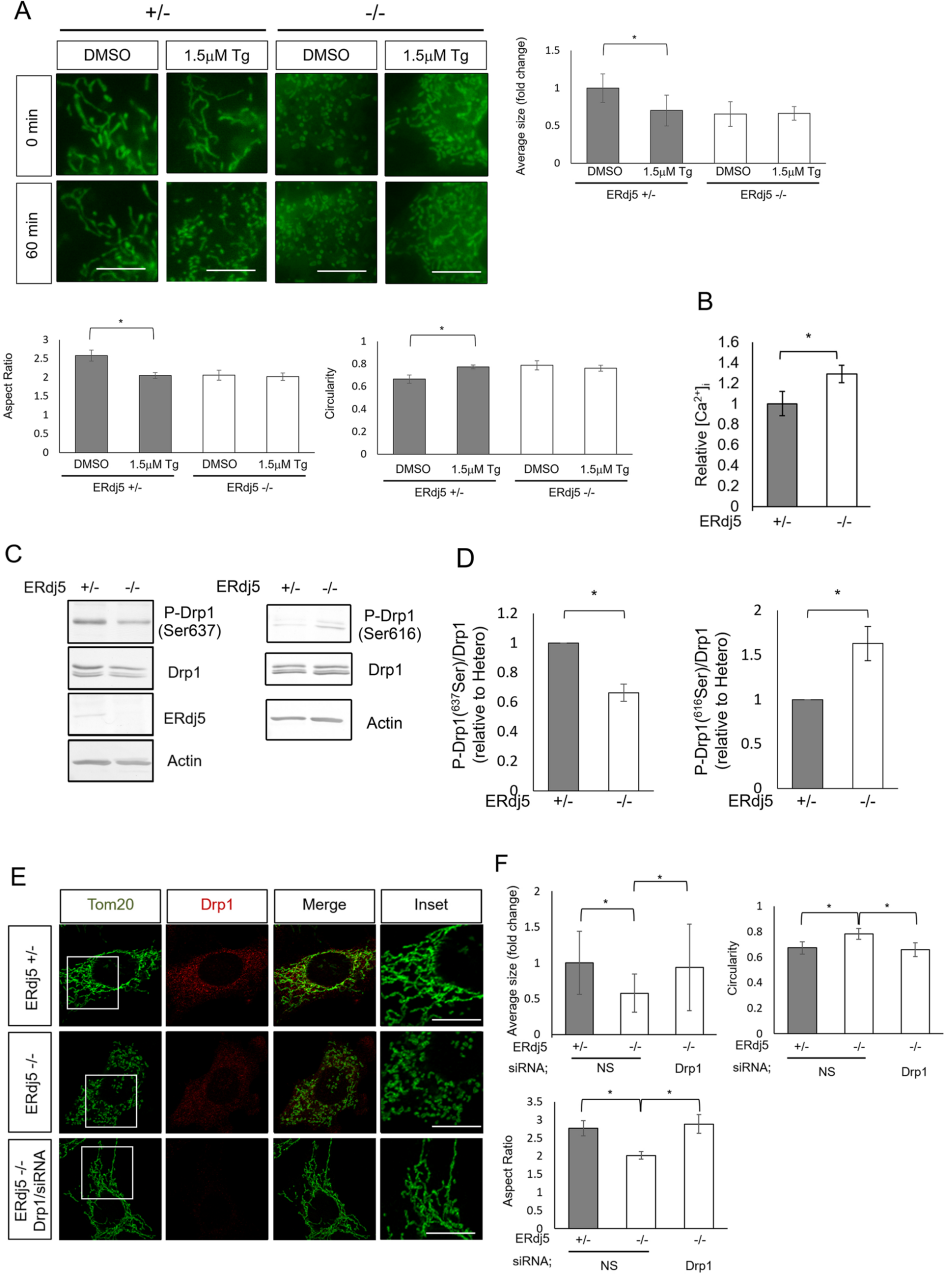
The deletion of ERdj5 activated Drp1 through high [Ca^2+^]_i_. (**A**) Cells were stained with MitoTracker Green (Green) in the presence or absence of 1.5 μM Tg, an inhibitor of the SERCA calcium pump on the ER membrane. The graph shows the relative extent, aspect ratio, and circularity of the mitochondria from MitoTracker Green, as analysed by ImageJ. *P < 0.05 by *t*-test. The results are reported as the means of 50 cells ± SDs. Scale bars = 10 µm (**B**) Measurement of relative [Ca^2+^]_i_ in ERdj5 ± or ERdj5−/− MEFs using Yellow Cameleon 3.6, a fluorescence resonance energy transfer (FRET)-based calcium imaging sensor. The [Ca^2+^]_i_ in each sample was determined in three independent experiments. *P < 0.05 by *t*-test. The results are reported as the means of 50 cells ± SDs. (**C**) The phosphorylation of Drp1 at Ser637 (P-Drp1) and Se616 (P-Drp1) in ERdj5 ± or ERdj5 -/- MEFs. Proteins in the cell lysates were separated by SDS-PAGE and immunoblotted with the indicated antibodies. (**D**) The intensities of the phosphorylated Drp1 bands in (C) were quantified by Image J and normalized by the amount of total Drp1 as indicated in the graphs. Data represent the mean ± SEM of 3 independent experiments. *p < 0.05 versus ERdj5 + /− MEF. (**E**) Mitochondrial morphology in ERdj5-deficient cells after Drp1 knockdown. Forty-eight hours after transfection with nonspecific or Drp1-specific siRNA, the cells were immunostained with anti-Tom20 (green) and anti-Drp1 (red). Insets show high-magnification views of the boxed areas. Scale bars = 10 µm (**F**) The average extent, circularity, and aspect ratio of mitochondria from Tom20 in the cells shown in (**E**). *P < 0.05 by *t* test. The results are reported as the means of 50 cells ± SDs.

Previously, we demonstrated that ERdj5 cleaved the disulfide bridge in the luminal loop of SERCA2b, a Ca^2+^ pump on the ER membrane, and promoted calcium uptake into the ER from the cytosol^20^. Deletion of ERdj5 prevented calcium uptake by SERCA2b and decreased [Ca^2+^]_ER_. Thus, we next examined the effect of [Ca^2+^]_i_ on the fragmentation of mitochondria. Treatment with Tg, an inhibitor of the SERCA family, was reported to prevent the uptake of [Ca^2+^]_ER_ via the SERCA pump and increase [Ca^2+^]_i_ due to leakage from the ER to the cytosol and release through IP3R or RyR. In the presence of 1.5 μM Tg, the number of fragmented mitochondria was increased in ERdj5 + /− MEF (Fig. 2A). The steady-state level of [Ca^2+^]_i_ in ERdj5-deficient cells was approximately 1.4 times higher than that in WT cells, as revealed by analysis with Yellow Cameleon 3.6 (YC3.6), a fluorescence resonance energy transfer (FRET)-based Ca^2+^ sensor (Fig. 2B). Ca^2+^ influx into mitochondria from the ER after IP3R stimulation by histamine was monitored by the mitochondria-localized Ca^2+^ sensor CEPIA2 mt. There was no significant difference in Ca^2+^ influx into the mitochondria between WT and ERdj5 KO cells (Fig. S2). Mitochondrial Ca^2+^ was measured by the mitochondria-localized Ca^2+^ probe Rhod-2. There was no significant difference between WT and ERdj5 KO cells (Fig. S3). Taken together, these results suggest that the deletion of ERdj5 causes the perturbation of Ca^2+^ levels in the ER and cytosol of mammalian cells but not in mitochondria.

### The fragmentation of mitochondria in ERdj5-deficient cells depends on Drp1 activation

Mitochondrial morphology is dynamic and changed by coordinated fission and fusion. To address the mechanism of [Ca^2+^]_i_-dependent mitochondrial fragmentation, we focused on the role of dynamin-related protein 1 (Drp1), a cytosolic GTPase involved in mitochondrial fission. cAMP-dependent protein kinase A (PKA) phosphorylates Ser637 in the GTPase effector domain (GED) of human Drp1. This phosphorylation inhibits mitochondrial fission by inhibiting the intramolecular interaction between the GTPase domain and GED domain of Drp1, GTPase activity, and eventually the mitochondrial recruitment of Drp1^26,27^. Conversely, calcineurin, an [Ca^2+^]_i_-dependent phosphatase, dephosphorylates phosphorylated Ser637 (Ser637 (P)) in Drp1 and stimulates Drp1 translocation to the mitochondrial membrane^28^.

In addition, Ser616 of Drp1 is phosphorylated by Calmodulin kinase II (CamKII), a calcium-dependent phosphatase. Phosphorylation of Ser616 in Drp1 promotes translocation of Drp1 to mitochondria and mitochondrial cleavage^29,30^.

To examine the activation of Drp1 in response to [Ca^2+^]_i_, we examined the ratio of phosphorylated vs total Drp1 using a specific antibody for Ser637(P) or Ser616(P). The results showed that phosphorylation of Ser637 was decreased and phosphorylation of 616 was increased in ERdj5−/− MEFs. (Fig. 2C,D). Next, we examined mitochondrial morphology in ERdj5-deficient cells after Drp1 knockdown (Fig. S4). Drp1 knockdown in ERdj5−/− cells restored the mitochondrial morphology from a fragmented morphology to a tube-like morphology (Fig. 2E,F). On the other hand, mammalian mitochondrial morphology was also reported to be regulated through the processing of OPA1 in mitochondrial inner membrane potential (ΔΨ-dependent manner^31^. However, we did not observe a significant difference in the processing of OPA-1 between ERdj5 ± and ERdj5-/- MEFs (Fig. S5). Additionally, we confirmed that the ΔΨ was no difference between ERdj5 ± and ERdj5−/− MEFs, which was examined with the JC-1 MitoMP detection kit (Fig. S6). This finding suggests that mitochondrial fission by the deletion of ERdj5 is independent of the dissipation of the ΔΨ.

### The sensitivity of ERdj5-deficient cells to apoptosis

**Figure 3:**
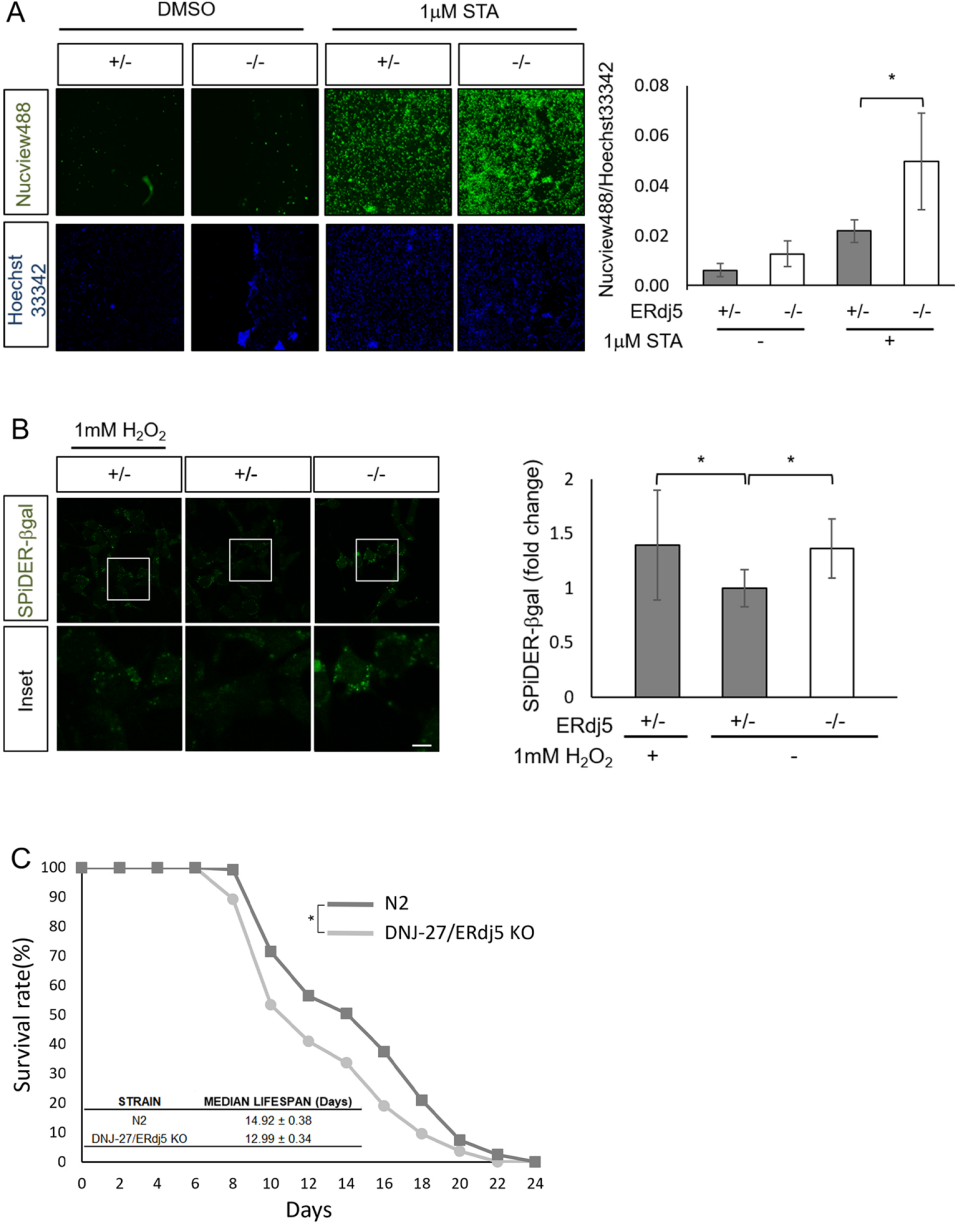
The physiological relevance of ERdj5 in the apoptosis, cellular senescence, and longevity of *C. elegans.* (**A**) Sensitivity to apoptosis in the presence of staurosporine (STA). One hour after incubation in the presence or absence of 1 μM STA, cells were stained with Hoechst 33,342 and NucView488, a substrate of Caspase-3. The ratio of NucView 488 fluorescence to Hoechst 33,342 staining was monitored in each cell. The results are reported as the means ± SDs of three replicates. (**B**) Imaging of senescence-associated β-galactosidase (SAβ-gal). Six hours after incubation in the presence or absence of 1 mM H_2_O_2_, the cells were stained with Hoechst 33,342 SPiDER-βGal at 37 °C for 30 min following the protocol. Insets show high-magnification views of the boxed areas. *P < 0.05 by *t* test. The results are reported as the means of 50 cells ± SDs and were determined in three experiments. Scale bars = 10 µm (**C**) The lifespan of DNJ-27/ERdj5-deficient worms. Surviving WT and DNJ-27/ERdj5 KO worms (RB1784 strain) were incubated at 20 °C and scored every other day. Survival curves and mean lifespans were analysed using OASIS2. n = 114–137 animals per strain, *P < 0.05 by *Rog-Rank* test.

Previous reports have revealed that aberrant mitochondrial fragmentation enhances sensitivity to apoptosis^27^. Treatment with staurosporine (STA), a protein kinase C inhibitor, is known to induce aberrant mitochondrial fragmentation and apoptosis^32^. We examined whether the deletion of ERdj5 would enhance the sensitivity of the cells to apoptosis in the presence of STA. To evaluate the sensitivity to apoptosis, we observed the activation of caspase-3 using the NucView 488 assay. The caspase-activated NucView-positive signal was increased in ERdj5−/− MEFs treated with STA (Fig. 3A). This suggests that ERdj5 deficiency caused cells to be more sensitive to apoptosis through mitochondrial fission.

### The deletion of ERdj5 induces phenotype with features of cellular senescence

A previous report showed that mitochondrial fission through the hyperactivation of Drp1 increases the production of reactive oxygen species (ROS) in the mitochondria and finally accelerates cellular senescence^33^. To examine whether ERdj5 is involved in cellular senescence, we investigated the activity of β-galactosidase, a marker of cellular senescence, using the SPiDER-βGal kit in ERdj5 ± and ERdj5-/- MEFs. As shown by this assay, the deletion of ERdj5 increased the fluorescence of SPiDER-βGal to the same extent as 1 mM H_2_O_2_ treatment for 6 h (Fig. 3B). Moreover, we assessed the involvement of ERdj5 in individual ageing with *C. elegans*. To monitor the lifespan of the worms, WT and DNJ-27/ERdj5 KO worms were incubated at 20 °C, and the number of surviving worms was scored every other day. The deletion of DNJ-27/ERdj5 slightly decreased the lifespan of the worms (Fig. 3C), consistent with the results regarding phenotype with the feature of cellular senescence (Fig. 3B). These results suggest the possibility that ERdj5 has important roles in mitochondrial homeostasis, which involves the maintenance of cellular senescence.

## Discussion

We have shown that the downregulation of ERdj5 causes aberrant mitochondrial fission, resulting in the production of fragmented mitochondria, and revealed its mechanism; the knockdown of ERdj5 caused an increase in [Ca^2+^]_i_ in mammalian cells, followed by the activation of Drp1, which assembles around the mitochondria and constricts both outer and inner mitochondrial membranes.

Another group showed that an ERdj5 orthologue in worms (DNJ-27) elicited a protective effect against proteotoxicity caused by aggregation in the cytosol^25^. Moreover, the authors showed that the deletion of DNJ-27 caused severe paralysis and slow mobility in neurodegenerative disease model animals. Here, we observed mitochondrial fission in the intestine in *C. elegans*. Cytosolic Ca^2+^ signalling is essential for the contraction of muscle and peristaltic motion of the intestines. Therefore, since calcium homeostasis in muscle and intestinal cells should be maintained more strictly than in other tissues, it is reasonable that intracellular calcium homeostasis in intestinal cells was significantly disrupted due to ERdj5 deficiency and that remarkable mitochondrial disruption was observed in intestinal cells. Furthermore, in this report, we showed that the downregulation of ERdj5 also caused aberrant mitochondrial fragmentation in mammalian cells. A previous report demonstrated that ER stress was strongly induced, especially in the salivary gland, by just the disruption of ERdj5. Interestingly, a recent study showed that the deletion of ERdj5 resulted in a Sjögren’s syndrome-like phenotype in mice. Sjögren’s syndrome induces salivary gland dysfunction that leads to xerostomia. Although the detailed mechanism is still unknown, salivary fluid secretion is prevented by abnormal calcium signals in salivary gland acinar cells^34^. Our conclusion that the downregulation of ERdj5 results in the perturbation of [Ca^2+^]_i_ is consistent with this pathogenetic mechanism of Sjögren’s syndrome.

In this report, we found that the steady-state [Ca^2+^]_i_ was approximately 1.4 times higher in ERdj5-deficient cells than in WT cells (Fig. 2B). Previously, we reported the redox-assisted regulation of SERCA2b mediated by ERdj5 by showing that the deletion of ERdj5 negatively regulated the pump function of SERCA2b and resulted in a low [Ca^2+^]_ER_^20^. Therefore, one of the causes by which ERdj5 deficiency increases the steady-state [Ca^2+^]_i_ is a decrease in SERCA2b pump function. This result is consistent with the inhibition of the SERCA2 pump with thapsigargin, and indeed, the prevented SERCA2b pump function causes mitochondrial fragmentation. However, it cannot be excluded the other possibility that the increase in [Ca^2+^]_i_ due to ERdj5 deficiency may change the release and leakage of Ca^2+^ from the ER to the cytosol, including the change in MAM formation. It would be interesting to examine if ERdj5 is involved in cellular Ca^2+^ dynamics in addition to regulating the SERCA2b pump. Previous report showed that STA treatment enhanced the release of Ca^2+^ through IP3R. This activation by STA increased the transport of Ca^2+^ into the mitochondria and caused depolarization of mitochondria, resulting in apoptosis^35^. As shown in Fig. 3A, the vulnerability to STA in ERdj5-deficient cells may be due to mitochondrial fragmentation and changes in Ca^2+^ dynamics by ERdj5 deficiency. Additionally, in Fig. 3B, we monitored oxidative stress in ERdj5-deficient cells using SPiDER-βgal. A previous report showed that oxidative stress under ER stress conditions prevents SERCA2 pump activity. Oxidative stress may further suppress the activity of SERCA2b and increase the [Ca^2+^]_i_^15^. Elevated [Ca^2+^]_i_ is caused by various factors, affecting various intracellular homeostasis, which can lead to a vicious cycle. It is currently difficult to conclude that the increase in cytosolic Ca^2+^ concentration due to ERdj5 deficiency is one cause.

In addition, we show that ERdj5 deficiency causes a steady increase in [Ca^2+^]_i_. However, transient increases in intracellular Ca^2+^ can make a more significant difference. For example, [Ca^2+^]_i_ changes dynamically during the cell cycle. During G1/S phase, Ca^2+^ is released from the ER to the cytosol through the IP3R channel, and extracellular Ca^2+^ also flows into the cells via the STIM1/Orai1 pathway^36^. After such a transient increase in [Ca^2+^]_i_, the delay in Ca^2+^ clearance from the cytosol in ERdj5-deficient cells may cause mitochondrial fission due to the activation of Drp1.

**Figure 4:**
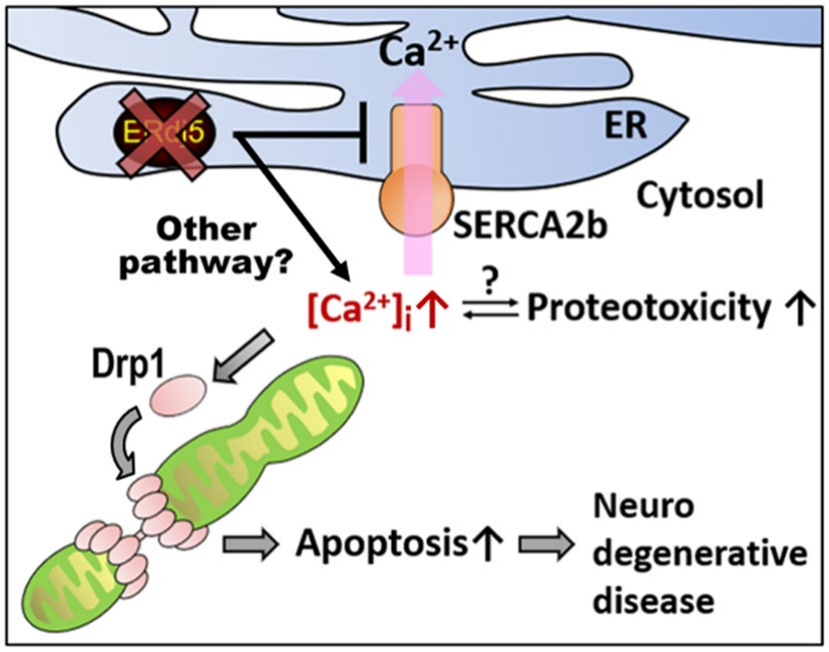
Dysfunction of the ER-resident reductase ERdj5 caused aberrant mitochondrial fragmentation due to the imbalance of intracellular Ca^2+^ homeostasis. The dysfunction of ERdj5 prevents Ca^2+^ uptake from SERCA2b and causes an imbalance in [Ca^2+^]_i_. Increased [Ca^2+^]_i_ constitutively activates Drp1, which is involved in mitochondrial fission. The calcium imbalance and abnormal mitochondrial fission caused by ERdj5 dysfunction may be responsible for intracellular proteotoxicity.

The knockdown of DNJ-27/ERdj5 was reported to cause severe paralysis in *C. elegans*^25^. The authors of this report confirmed that DNJ-27/ERdj5 overexpression prevented paralysis in neurodegenerative model worms. Previous studies suggested a close correlation between some neurodegenerative diseases and the perturbation of intracellular calcium homeostasis^37–39^; the impairment of ER membrane permeability by the accumulation of amyloid proteins was reported to increase intracellular free Ca^2+^ levels^37^, and ROS caused by the accumulation of amyloids preferentially damaged some ion pumps, including SERCA family proteins, resulting in an increase in [Ca^2+^]_i_^40^. Our data suggest that the decrease in cell viability under higher [Ca^2+^]_i_ conditions caused by the deletion of ERdj5 could enhance sensitivity to proteotoxicity (Fig. 4). Hence, the ERdj5-mediated maintenance of Ca^2+^ homeostasis in both the ER and cytosol might be critical for a potential therapeutic strategy for neurodegenerative disease. Other groups have also noted the possibility that ERdj5 could serve as a novel chemotherapeutic target for cancer; the retinoid analogue fenretinide was shown to be a cancer-preventive and chemotherapeutic drug. Unlike most retinoids, fenretinide induces apoptosis in vitro^24^. The knockdown of ERdj5 by RNAi in neuroectodermal tumour cells and melanoma cells increased the apoptotic response to fenretinide. While the collapse of ER protein quality control due to the downregulation of ERdj5 was described to enhance the sensitivity to apoptosis in this report, we would also like to note the possibility that the perturbation of calcium by ERdj5 caused proteostasis instability, leading to apoptosis in cancer cells.

The mammalian ER contains nearly 20 members of the protein disulfide isomerase (PDI) family, including ERdj5, which together constitute a redox environment that maintains ER homeostasis. Although a luminal cysteine pair in SERCA2b is cleaved by ERdj5 via its reductase activity, the functional redundancy of ERdj5 is well unknown. A previous report showed that some ERdj5 downregulation-mediated phenotypes, including general paralysis and slow motility, were more clearly observed in *C. elegans* than in ERdj5-deficient mice. This suggests that some PDI family proteins or other mechanisms may complement the functions of ERdj5 in mammals but not in *C. elegans*. Therefore, further studies are needed to understand the maintenance of ER homeostasis through the redox network centred on ERdj5.

## Methods

### Plasmids and siRNA

Mouse ERdj5/WT and ERdj5/AA plasmids were constructed as described previously^4^. YC3.6 was a gift from Takeharu Nagai (Osaka University). CEPIA2_mt_ was purchased from Addgene. siRNAs against the following target sequences from mouse DRP1 and human ERdj5 were synthesized by Invitrogen: mouse DRP1: 5′-GGUGGUGCUAGGAUUUGUUAUAUUU-3′, 5′-UUCAGCAUUCCUCUCCAGUUGCCUG-3′, human ERdj5: 5′-AGUGGCAUGUUGGACGGCCUUGUUA-3′, 5′-UAACAAGGCCGUCCAACAUGCCACU-3′.

### Cell culture and transfection

HeLa cells were kindly provided by Dr. Tamotsu Yoshimori (Osaka University, Japan). MEFs, HeLa cells (HeLa Kyoto strain), and HEK293 cells were maintained in DMEM (Thermo Scientific) containing 10% fetal bovine serum (Sigma-Aldrich), 100 U/mL penicillin, and 100 μg/mL streptomycin. Plasmids were transfected into MEFs using Lipofectamine LTX (Invitrogen). In HeLa cells, plasmids were transfected by using PEI MAX (Polysciences). The amount of DNA was adjusted to obtain equivalent expression levels of the introduced proteins in each experiment. siRNAs were transfected into MEFs using Lipofectamine RNAiMax (Invitrogen).

### Antibodies

The following antibodies were used in this study: anti-FLAG (Sigma-Aldrich), rabbit polyclonal anti-Myc (MBL Life Science), mouse monoclonal anti-ERdj5 (SANTA CRUZ), mouse monoclonal anti-DLP1 (BD Bio Science), rabbit polyclonal anti-Tom20 (Abcam), rabbit polyclonal anti-phospho-DRP1 (Ser637) (Cell Signalling Technology), rabbit monoclonal anti-OPA1 (Cell Signalling Technology) and mouse monoclonal anti-Actin (Merck Millipore). Alkaline phosphatase AffiniPure goat anti-mouse IgG (H + L) polyclonal antibody (Jackson ImmunoResearch), alkaline phosphatase AffiniPure goat anti-rabbit IgG (H + L) polyclonal antibody (Jackson ImmunoResearch), Alexa Fluor 488-conjugated goat anti-mouse IgG (H + L) highly cross-adsorbed secondary polyclonal antibody (Thermo Scientific), Alexa Fluor 555-conjugated goat anti-mouse IgG (H + L) highly cross-adsorbed secondary polyclonal antibody (Thermo Scientific), Alexa Fluor 488-conjugated goat anti-rabbit IgG (H + L) highly cross-adsorbed secondary polyclonal antibody (Thermo Scientific), and Alexa Fluor 546-conjugated goat anti-rabbit IgG (H + L) highly cross-adsorbed secondary polyclonal antibody (Thermo Scientific) were also used.

### Immunoblotting

Samples were separated by SDS-PAGE using a 7.5% acrylamide gel and transferred to nitrocellulose membranes (GE Healthcare) for 1 h at 100 V and 4 °C. The membranes were treated with Blocking One (Nacalai Tesque) for 30 min at room temperature and then incubated sequentially with primary and secondary antibodies diluted in Blocking One in PBST. Detection was performed with SIGMAFAST BCIP/NBT (Sigma-Aldrich).

Uncropped blots are shown in (Fig. S7).

### Fluorescence microscopy

Fluorescence microscopy was performed using an SZX16 stereomicroscope system equipped with an SDF PLAPO 0.5 × PF or SDF PLAPO 1 × PF (Olympus Corp.) objective lens, a DeltaVision Elite fluorescence microscope (GE Healthcare Life Science) equipped with a 60 × PlanAPO oil immersion objective lens (Olympus; NA 1.42), or an LSM700 (Zeiss) confocal laser scanning microscope equipped with an α Plan-APOCHROMAT 63x/1.4 oil objective lens. For immunofluorescence observations, the cells were fixed for 15 min at room temperature with 4% paraformaldehyde. Then, the cells were permeabilized with 0.5% Triton X-100 in PBS for 15 min at room temperature. After permeabilization, the cells were blocked using 5% BSA containing PBS (blocking buffer) for 1 h and incubated with 2.5 μg/ml anti-Tom20 (Abcam) and 1:200 anti-Myc (MBL Life Science) antibodies in blocking buffer for 1 h. After washing the cells with PBS, the cells were incubated with secondary anti-IgG antibodies (Invitrogen) in blocking buffer for 1 h. Mitochondrial circularity, size, and number were measured with Fiji/ImageJ software. For MitoTracker staining, cells were incubated with 100 ng/ml MitoTracker Red CMXRos or MitoTracker Green FM (Thermo Scientific) in DMEM for 30 min at 37 °C in 5% CO_2_. For observation, cells were washed two times with PBS, and the medium was replaced with fresh DMEM. To observe the mitochondria in worms, the worms were stained with 1 μg/ml MitoTracker Red (Thermo Scientific) in M9 buffer for 1 h at 20 °C in the dark. Then, the worms were washed with M9 buffer three times and placed on a 3% agarose gel pad.

### Ca^2+^ imaging in live cells

Twenty-four hours after the transfection of YC3.6 into HeLa cells, the FRET ratio of YC3.6 was observed with a two-photon microscope equipped with a PlanApoN 60X/1.42 objective lens (Olympus). The Ca^2+^ sensor YC3.6 was excited with a 438-nm cyan fluorescent protein (CFP) excitation filter line of the LED. CFP (475 nm) and FRET-dependent Venus (548 nm) emission spectra were collected using the 458-nm primary dichroic mirror of the microscope. For time-lapse analysis, fluorescence images were acquired at approximately 0.5 frames/second for 33 min and reconstructed using Fiji/ImageJ software. To estimate the intracellular calcium ion concentration ([Ca^2+^]_i_), we first measured the steady-state fluorescence ratio (R) between CFP and YFP with HBSS containing 1.26 mM CaCl_2_^41^. Then, the maximum fluorescence ratio R_max_ was obtained by the fluorescence ratio of YC3.6 after the medium was replaced with HBSS containing 20 mM CaCl_2_ and 1 mM ionomycin. The minimum fluorescence ratio R_min_ was obtained after depleting the intracellular calcium ions by washing the cells with HBSS containing 1 mM EGTA, 5 mM MgCl_2_, and 1 mM ionomycin. Finally, [Ca^2+^]_i_ was calculated using the following equation in which the K'd value of YC3.6 and the Hill coefficient n were to be 0.25 and 1.7, respectively.

1 $$   \left[ {{\text{Ca}}^{{{\text{2}} + }} } \right]_{{\text{i}}}  = {\text{K'd}}\left[ {\left( {{\text{R}} - {\text{R}}_{{{\text{min}}}} } \right)\left( {{\text{R}}_{{{\text{max}}}}  - {\text{R}}} \right) - {\text{1}}} \right]^{{({\text{1}}/{\text{n}})}}     $$

The CEPIA2_mt_ fluorescence in HEK 293 cells was monitored with a Varioskan LUX multi-plate reader (Thermo Scientific) with 487-nm excitation and 508-nm emission wavelengths.

Analysis of mitochondrial Ca^2+^ levels was performed as previously described.

Briefly, cells were loaded with 0.8 mM Rhod-2 AM (Molecular Probes), 100 nM MitoTracker Green FM (Thermo Scientific) and 0.05% Pluronic F-127 (Molecular Probes) in DMEM containing 5 mM glucose for 30 min. Cells were washed once with recording medium [0.05% Pluronic F-127, 1.25 mM Probnecid, 1 mM CaCl_2_ in HBSS], followed by incubation at room temperature for 60 min in recording medium. Culture medium was changed to recording medium, and the cells were exposed to either 20 mM CCCP or 1 mM EGTA. Fluorescence signals were observed with an LSM700 (Zeiss) confocal laser scanning microscope. Fluorescence images labeled with Rhod-2 were collected using an excitation wavelength of 555 nm. Rhod-2 fluorescence was detected using single excitation and emission filters. Quantification was performed in the area where Rhod-2 and Mito-tracker green co-localized.

### Measurement of mitochondrial membrane potential

The cells were stained with JC-1 following the protocol. JC-1 was excited at 405 nm. The wavelength of JC-1 fluorescence emission changes from 555 to 488 nm in response to mitochondrial depolarization. The ratio of emission at 488 nm to that at 555 nm was evaluated as an index of mitochondrial depolarization. Fluorescence signals were observed with an LSM700 (Zeiss) confocal laser scanning microscope.

### Apoptotic activity assay

After treatment with 1 μM STA (Wako) for 1 h, the cells were incubated with 5 μM NucView 488 (Biotium Inc), a fluorescence-conjugated substrate of Caspase-3, and 3 μM Hoechst 33,342 (Thermo Scientific) for 1 h. After washing the cells with HBSS three times, fluorescence was observed with a Varioskan LUX multi-plate reader (Thermo Scientific) with excitation wavelengths of 488 nm (Nucview 488) or 361 nm (Hoechst 33,342) and emission wavelengths of 520 nm (Nucview 488) or 497 nm (Hoechst 33,342).

### Senescence-associated β-galactosidase staining

SPiDER-βGal was purchased from Dojindo Laboratories. After MEFs had been harvested on glass-bottom dishes (Matsunami), the cells were stained with SPiDER-βGal and Hoechst 33,342 (Thermo Scientific) at 37 °C for 30 min following the protocol of the SPiDER-βGal kit. Imaging was performed with an LSM 700 confocal microscope (Zeiss) with an excitation wavelength of 488 nm (SPiDER-βGal) or 361 nm (Hoechst 33,342) and an emission wavelength of 550 nm (SPiDER-βGal) or 497 nm (Hoechst 33,342).

### Lifespan assay

The embryos in hermaphroditic worms were synchronized by treatment with alkaline hypochlorite and allowed to develop for 3 days. Adult worms were incubated at 20 °C, transferred to new nematode growth medium (NGM) plates and scored every other day. A worm was judged as dead when its pharyngeal pumping ceased, and the worm did not respond to prodding with a platinum wire. Lifespan curves were plotted using Kaplan–Meier survival curves and analysed using log-rank tests. Mean lifespans were plotted and analysed using OASIS 2 (Online Application for Survival Analysis 2; https://sbi.postech.ac.kr/oasis2)^42^.

## Supplementary Information

Supplementary Information.
