# Supplementary material for: Ca2+ imbalance caused by ERdj5 deletion affects mitochondrial fragmentation
Source: Sci Rep. 2021 Nov 2;11:20772. doi: 10.1038/s41598-021-99980-9 (PMC8563984; doi:10.1038/s41598-021-99980-9)
Supplement: Supplementary file 1 — Supplementary Information. [file 41598_2021_99980_MOESM1_ESM.pdf]

Ca^{2+} imbalance caused by ERdj5 deletion affects mitochondrial fragmentation

Riyuji Yamashita[1], Shohei Fujii[1], Ryo Ushioda[1, 2*] & Kazuhiro Nagata[1, 2, 3*]

[1]Laboratory of Molecular and Cellular Biology, Faculty of Life Sciences, Kyoto Sangyo University, Kyoto 603-8555, Japan, [2]Institute for Protein Dynamics, Kyoto Sangyo University, [3]JT Biohistory Research Hall, Murasaki Town 1-1, Takatsuki City, Osaka 569-1125, Japan* Correspondence and requests for materials should be addressed to R.U. (ryo3ussy3@cc.kyoto-su.ac.jp) or K.N. (email: nagata@cc.kyoto-su.ac.jp)

Supplementary Figure 1

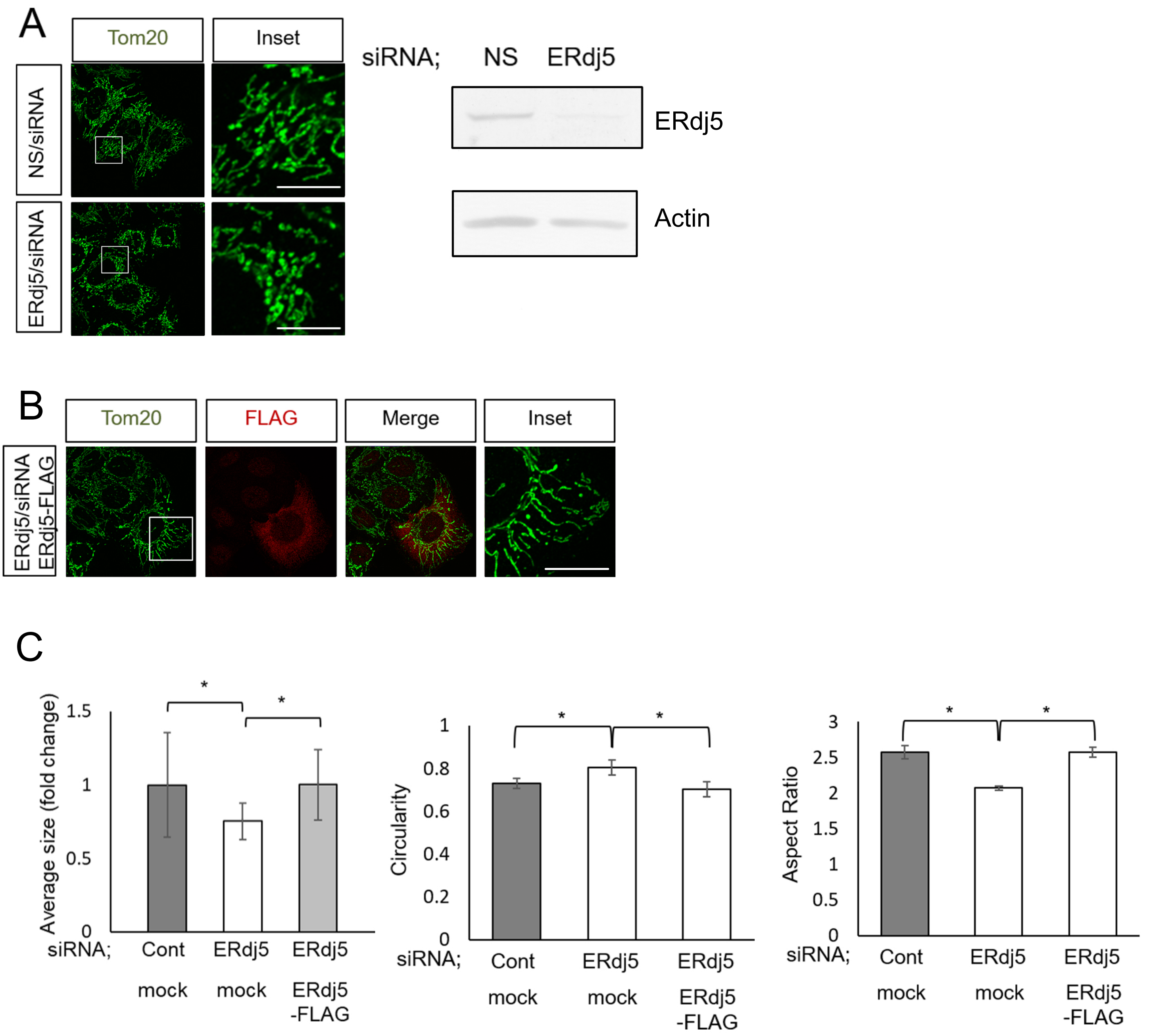

Supplementary Figure 1 ERdj5 knockdown caused mitochondrial fragmentation in mammalian cells. (A) Mitochondrial morphology of HeLa cells after ERdj5 knockdown. Forty-eight hours after transfection with nonspecific or ERdj5-specific siRNA, the cells were immunostained with anti-Tom20 antibody (green). Insets show high-magnification views of the boxed areas. Scale bars=10 μm. The right figure shows Expression of ERdj5 in scrambled (NS) and ERdj5 siRNA-transfected MEFs. Proteins in the lysates were separated by SDS-PAGE and immunoblotted with anti-ERdj5 antibody. **(B)** Twenty-four hours after the transfection of ERdj5-knockdown HeLa cells with mouse ERdj5-FLAG, the cells were immunostained with anti-Tom20 (green) and anti-FLAG (red) antibodies. Insets show high-magnification views of the boxed areas. Scale bars=10 μm **(C)** The relative average size, circularity, and aspect ratio of fluorescence from Tom20 in the cells shown in (A) and (B). *, $P < 0.05$ by t-test. The results are reported as the means of 50 cells± SDs.

Supplementary Figure 2

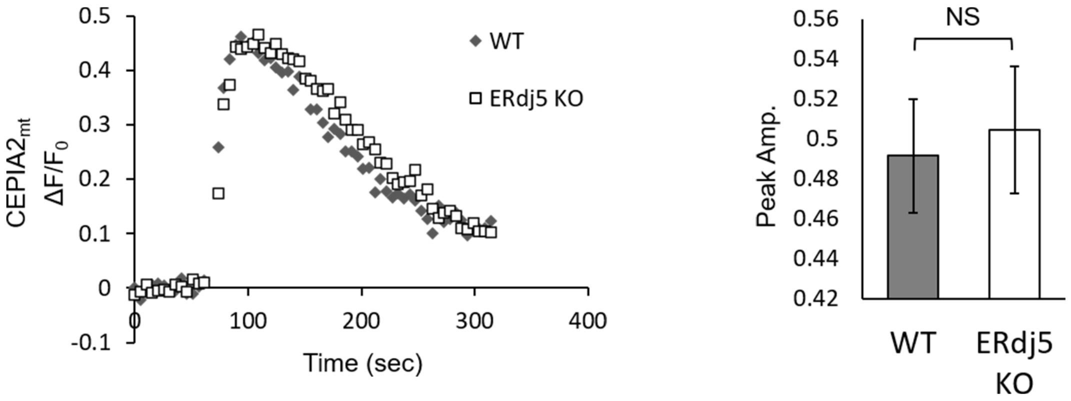

Supplementary Figure 2 Mitochondrial calcium concentration after treatment with histamine. Mitochondrial Ca^{2+} dynamics in response to histamine were measured with CEPIA2mt. The amplitude was defined as the maximum decrease in ⊿F/F0 within 30 seconds after 300 μM histamine stimulation.

Supplementary Figure 3

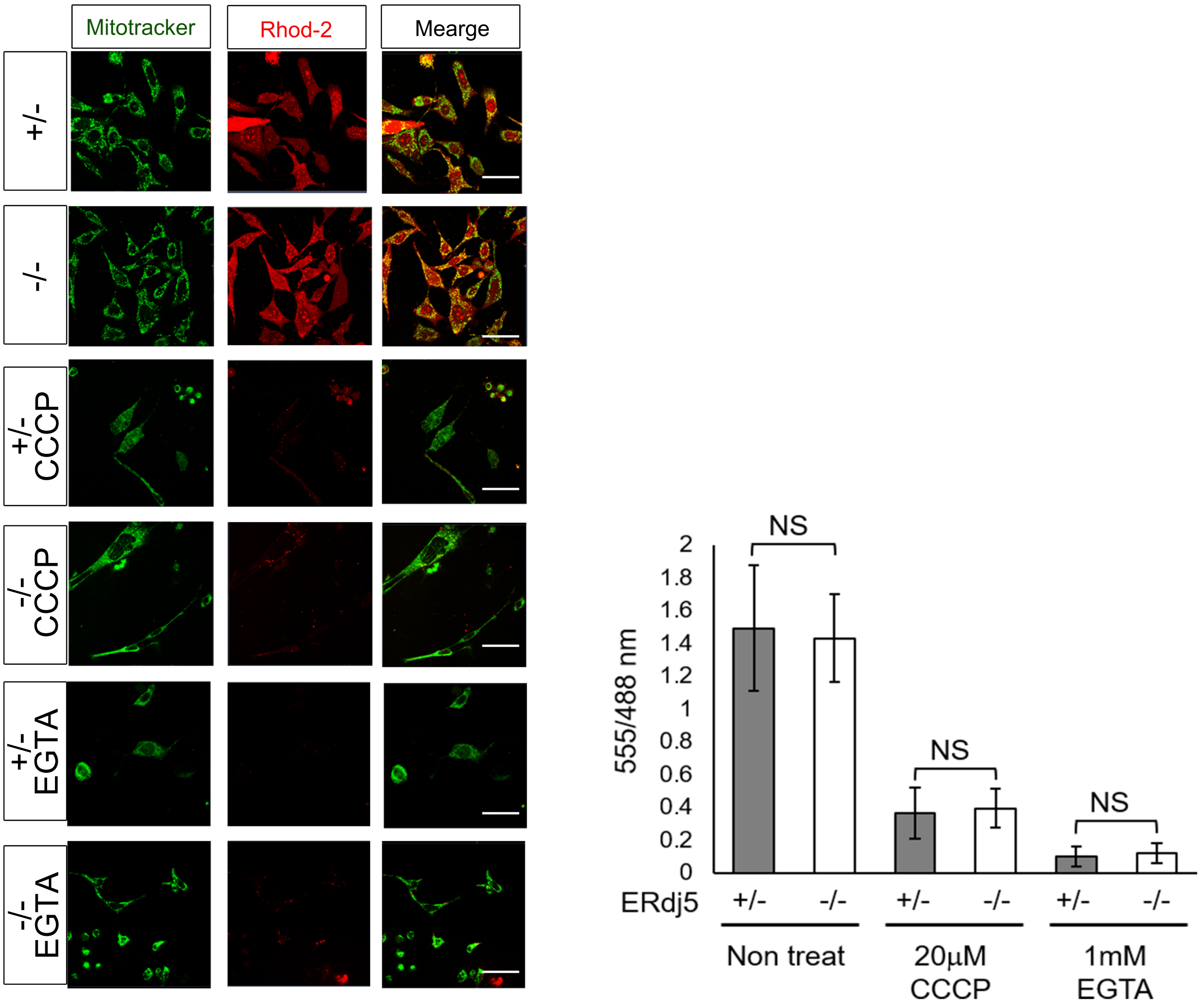

Supplementary Figure 3 Mitochondrial calcium in ERdj5 +/- and -/- cells.

Quantification of Rhod-2 fluorescence in ERdj5 +/- or -/- MEF cell.

ERdj5 +/- and -/- MEF cell were treated with 20[illegible]M CCCP or 1mM EGTA for 1hour. For quantification, we measured the fluorescence ratio in the area where Rhod-2 and Mito-tracker green co-localized. Scale bars =50 µm *, $P < 0.05$ by t-test. The results are reported as the means of 50 cells± SDs.

Supplementary Figure 4

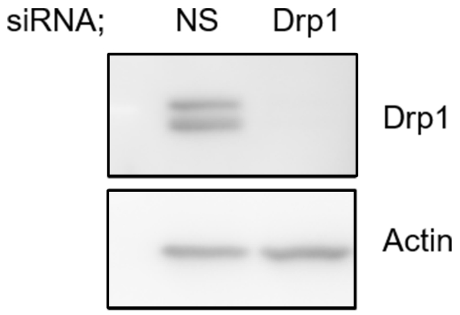

Supplementary Figure 4 Knockdown efficiency of Drp1 siRNA.
Expression of Drp1 in scrambled (NS) and Drp1 siRNA-transfected MEFs. Proteins in the lysates were separated by SDS-PAGE and immunoblotted with anti-Drp1 antibody.

Supplementary Figure 5

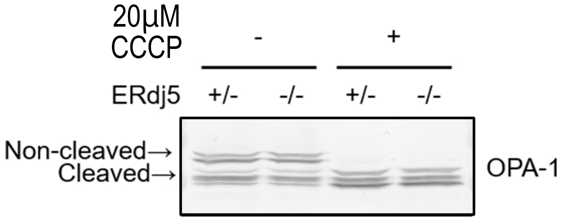

Supplementary Figure 5 The cleavage of OPA-1 in ERdj5+/- or ERdj5 -/- MEFs

The cells were incubated in the presence or absence of 20 μM CCCP, a protonophore, for 1 hour. Proteins in the lysates were separated by SDS-PAGE and immunoblotted with anti-OPA1 antibody.

Supplementary Figure 6

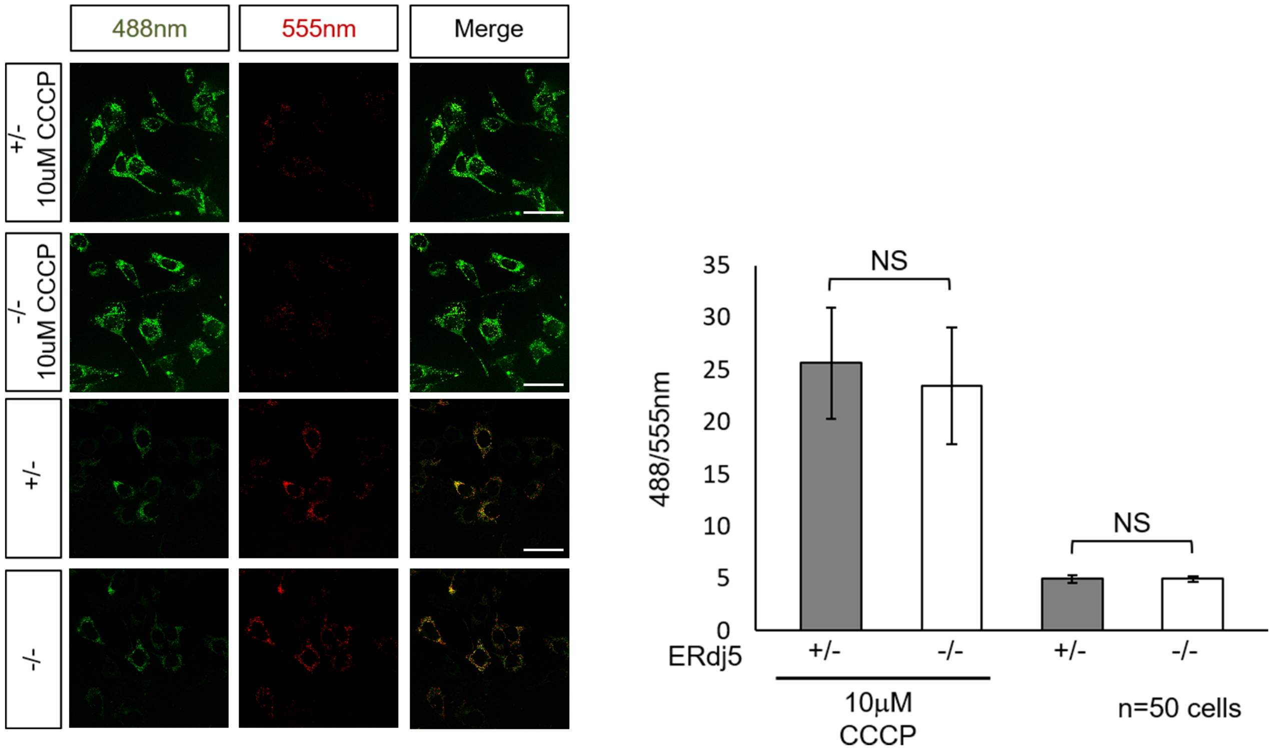

Supplementary Figure 6 The deletion of ERdj5 did not affect the mitochondrial inner membrane potential.

After incubation in the presence or absence of CCCP, cells were stained with JC-1 following the protocol. The wavelength of JC-1 fluorescence emission changed from 555 nm to 488 nm in response to mitochondrial depolarization. The ratio of emission at 488 nm to 555 nm is shown in the graph on the right. Scale bars=50 μm *, $P < 0.05$ by t-test. The results are reported as the means of 50 cells± SDs.

Supplementary Figure 7

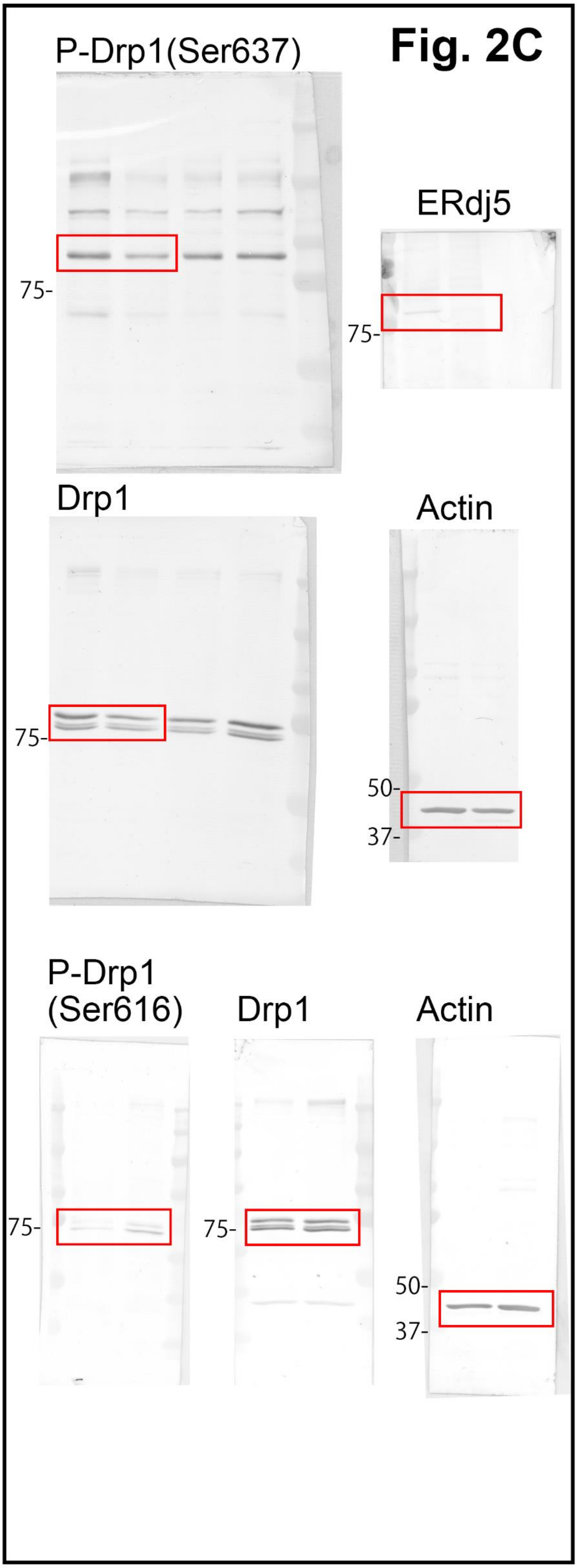

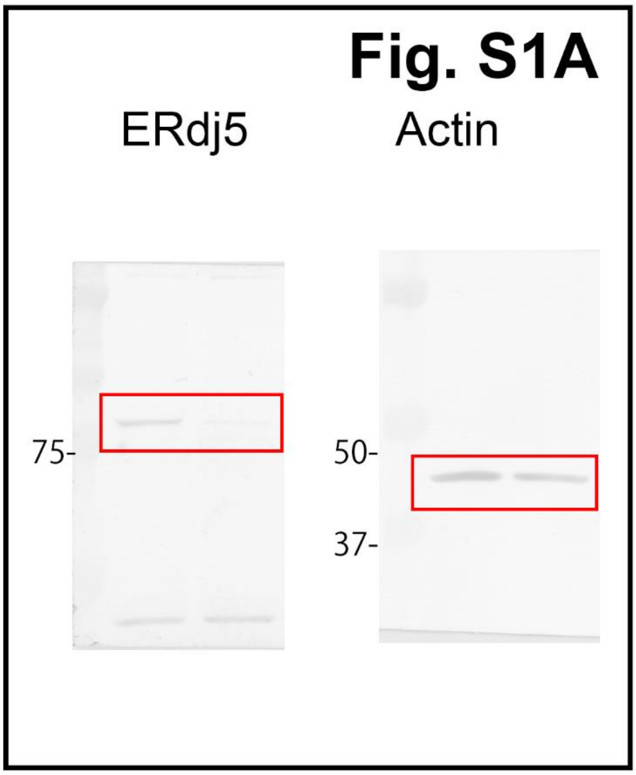

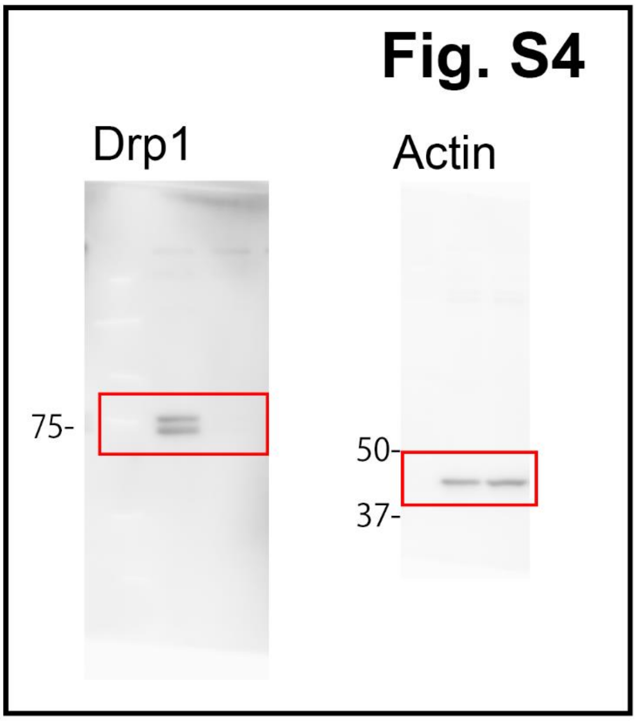

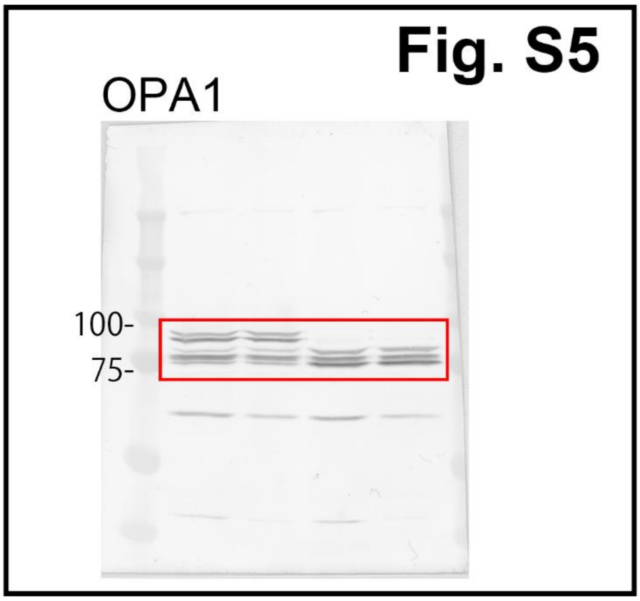

Supplementary Figure 7 Uncropped blots of figures.
